# The Oral Microbiome of Peri-Implant Health and Disease: A Narrative Review

**DOI:** 10.3390/dj12100299

**Published:** 2024-09-24

**Authors:** Natalia de Campos Kajimoto, Yvonne de Paiva Buischi, Mansour Mohamadzadeh, Peter Loomer

**Affiliations:** 1 Department of Periodontics, The University of Texas Health Science Center at San Antonio, San Antonio, TX 78229, USA; kajimoton@uthscsa.edu (N.d.C.K.); buischiy@uthscsa.edu (Y.d.P.B.); 2 Department of Microbiology, Immunology & Molecular Genetics, The University of Texas Health Science Center at San Antonio, San Antonio, TX 78229, USA; zadehm@uthscsa.edu

**Keywords:** microbiota, peri-implantitis, dental implants, dysbiosis

## Abstract

Peri-implantitis disease has increased significantly over the last years, resulting in increased failure of implants. Many factors may play a role in implant complications and failure, including ones related to the oral microbiota. This literature review aims to summarize the current knowledge of microbiome of implants in health and disease, focusing not only on the presence/absence of specific microbiota or on their relative abundance, but also on their phenotypic expression and their complex relationships with the host. The authors examined the MEDLINE database and identified key topics about peri-implant oral microbiome in health and disease. The peri-implant microbiome differs from that of the tooth, both in health and disease, as they are structurally and chemically different. The adhesion and formation of the peri-implant biofilm can be affected by the surface energy, topography, wettability, and electrochemical charges of the implant surface. In addition, the morphogenesis of the tissues surrounding the dental implant also differs from the tooth, making the dental implant more susceptible to bacterial infection. This interplay between the microbiome and the host immune system in peri-implant infections still needs to be elucidated.

## 1. Introduction

Osseointegrated dental implants are the gold standard therapy for the replacement of lost or missing teeth. Despite their mostly successful outcomes and an elevated long-term survival, some mechanical and biological complications can occur [1,2]. The most frequent dental implant biological complications are peri-implant mucosa inflammation (peri-implant mucositis), followed by progressive bone loss around the dental implant (peri-implantitis) [2,3,4,5,6]. Peri-implantitis is regarded as the leading cause of implant loss [7]. The prevalence of peri-implant disease has been extensively studied [8,9,10,11], but the ranges vary extensively in the literature, due to variations in definition and clinical measurements used to detect peri-implant diseases [12]. A recent systematic review with meta-analysis showed a prevalence of peri-implantitis of 19.53% and 12.53% at patient and implant level, respectively [11].

The etiology of peri-mucositis and peri-implantitis is multifactorial, involving peri-implant biofilm deposition [2,3,4,5,6], the host response to biofilm [13,14,15,16,17], and environmental factors, such as systemic disorders, smoking, and iatrogenic dentistry [14,15,16,17,18,19,20]. However, the etiology of peri-implantitis is not fully understood and is still being investigated [21,22].

The presence of a pathogenic peri-implant biofilm has been described as the main risk indicator for peri-implant mucositis and peri-implantitis [18,23,24,25]. Whether specific pathogens play a role in initiating peri-implant diseases is not known, but there are numerous studies evaluating this phenomenon [23,26]. An accumulation of peri-implant biofilm in itself might be enough to initiate peri-implant mucositis [23,27,28,29], but not all peri-implant mucositis will develop into peri-implantitis. It appears that dysbiotic biofilm containing specific bacterial signatures is enough to instigate progressive bone loss [23]. However, these specific bacteria signatures have not been fully elucidated [23,26]. This knowledge would enhance not only our understanding of the peri-implantitis etiopathogenesis, but would also improve microbiological diagnostic procedures leading to patient-personalized therapies.

The objective of this literature review is to present a summary of the existing knowledge of the microbiome of implants in health and disease, focusing not only on the absence/presence of specific microbiota or on their relative abundance, but also on their phenotypic expression and their intricate interactions with the host.

## 2. Methodology

To assure a high-quality review process, the SANRA (Scale for the Assessment of Narrative Review Articles) was used [30]. SANRA comprises the subsequent topics: (1) justification of the review’s importance, (2) description of concrete objectives of the review, (3) methodology of the literature search, (4) referencing, (5) scientific reasoning, and (6) adequate data reporting [30].

To organize and select articles related to peri-implant oral microbiome, four authors (NCK, YPB, MZ and PML) independently reviewed manuscripts indexed in the MEDLINE database and accessible via PubMed. To determine which subjects should be included in this narrative review, the authors identified key topics about peri-implant oral microbiome that included the following: oral microbiome/microbiota of implants in health and disease (peri-implant mucositis and peri-implantitis), bacteria phenotypic expression and relationship with the host, and comparison of peri-implantitis and periodontitis oral microbiome/microbiota.

The search strategy used in this narrative review consisted of relevant and updated literature associated with peri-implant oral microbiome/microbiota. The authors (NCK, YPB, MZ and PML) took into consideration all peer-reviewed studies in English, including randomized controlled trials, clinical trials, case series, case reports, observational studies (case control studies/cross-sectional studies/cohort studies), meta-analyses, systematic review, narrative reviews, and other types of reviews. For this review, the authors searched PubMed until 31 March 2024 using the following MeSH terms: (“oral biofilm”), (“oral microbiome”), (“oral microbiota”), (“peri-implant healthy” AND “oral microbiome”), (“peri-implant healthy” AND “oral microbiota”), (“mucositis peri-implant” AND “oral microbiome”), (“mucositis peri-implant” AND “oral microbiota”), (“peri-implantitis” AND “oral microbiome”) and (“peri-implantitis” AND “oral microbiota”). The authors reviewed selected articles according to topic relevance. The reference lists of selected manuscripts were also revised. Relevant titles identified during this process led to further examination of the corresponding articles. If these articles provided additional pertinent information, they were subsequently included in the review.

## 3. Peri-Implant Supramucosal and Submucosal Definition and Standardization

**Figure 1:**
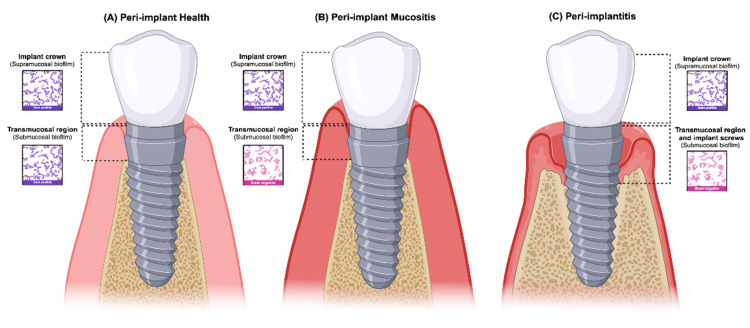
Classification of the peri-implant biofilm in health and disease and the type of microbiota associated with each condition. (**A**) Peri-implant health: the supramucosal biofilm is located on the implant crown and the submucosal biofilm is located in the transmucosal region. Both supramucosal and submucosal peri-implant biofilm support a unique microbiota dominated by facultative Gram-positive bacteria (peri-implant sulcus); (**B**) peri-implant mucositis: the supramucosal biofilm is located in the implant crown and the submucosal biofilm is located in the transmucosal region. In peri-implant mucositis, a shift occurs in the submucosal microbiota, harboring a high number of anaerobic Gram-negative bacteria (peri-implant pockets); (**C**) peri-implantitis: the supramucosal biofilm is located on the implant crown and the submucosal biofilm is located in the transmucosal region and implant screws. In peri-implantitis, the submucosal microbiota hosts abundant anaerobic Gram-negative species (peri-implant pockets). Created with BioRender.com (License number: bAK273B6HQL).

Although there is an assumption of close similarity of peri-implant supramucosal and submucosal biofilm with the tooth supragingival and subgingival biofilm, as per our knowledge, a definition for the peri-implant supramucosal and submucosal biofilm has not been described yet. The definitions adopted in this manuscript were based on normal implant health and peri-implant disease (peri-implant mucositis and peri-implantitis). The peri-implant biofilm in healthy implants is described as supramucosal when the biofilm is above the peri-implant mucosal margin (crown), and as submucosal when the biofilm is located below the peri-implant mucosal margin (transmucosal region) (Figure 1A). In peri-implantitis cases, the implant screws will also be colonized, and the biofilm will be classified as supramucosal or submucosal, depending on where the peri-implant mucosa margin is located (presence or not of mucosa recession) (Figure 1C). Therefore, the terms “supramucosal” and “submucosal” [31] should be utilized when referring to dental implants. For future peri-implant microbiome studies, these terms should be standardized as supramucosal and submucosal peri-implant biofilm.

## 4. What Do We Know about the Peri-Implant Biofilm?

In the oral cavity, the biofilm is a complex microbial community that adheres to the tooth and implant surfaces [32,33]. This biofilm has a high microbial cell density [34] and is encased within a protective extracellular matrix that protects microorganisms from a hostile environment, allowing for the maintenance of a balanced ecosystem [35,36,37]. Microorganisms interact with each other through “quorum sensing” [38], and are distributed in the mouth according to region, condition, and their metabolic and biochemical features [7]. The oral microbiome is functionally and structurally organized, meaning that the microbial population properties are more than the addition of the components contained within it [33,39,40]. In this scenario, the community structures and the bacterial co-occurrence networks need to be understood, to clarify the peri-implantitis effect on the oral microbiome [33].

### 4.1. Peri-Implant Biofilm Formation and Colonization

Salivary proteins adhere to the implant surface as soon as saliva touches it, forming the acquired salivary pellicle [41]. Initial bacterial adhesion is promoted by a rich protein structure formed on the implant surface, which provides receptors for adhesins from the cell surface of all oral bacterial species [42]. The acquired salivary pellicles formed on the tooth and dental implant are not identical. In vitro studies showed that the acquired salivary pellicle formed on titanium surfaces includes α-amylase, high-molecular-weight mucins, proline-rich proteins and secretory IgA. Molecules frequently detected on dental enamel, such as low-molecular-weight mucins and cystatins, were not found in the pellicle formed on the titanium surface [43].

Similarly to the natural dentition, microorganisms will colonize the implant surfaces exposed to the oral cavity [7,32,44,45]. Initial adhesion starts in the areas where bacteria are protected from shear forces [5,46,47]. It is activated by electrostatic, van der Waals and hydrophobic forces, which move the cells close to the implant surface covered by acquired salivary pellicle. After bacteria attach to proteins of the acquired salivary pellicle, an irreversible adherence becomes effective [5,48,49,50]. Later, intercellular electrostatic interconnections mediated by surface proteins and cell-wall proteins cause bacteria to gather jointly, forming microcolonies. This interchange also leads to bacterial co-aggregation and peri-implant biofilm deposition [32]. The variety of the microbial community increases as the peri-implant biofilm matures [5,48,49,50]. Lastly, nucleases and proteases are engaged in biofilm dispersal, through which cells move from the biofilm to join a planktonic stage [32].

Early stages of the peri-implant biofilm formation, facultative Gram-positive cocci, rods, nonmotile bacilli, and a restricted number of Gram-negative anaerobic species predominate on the supramucosal and submucosal peri-implant biofilm [31,32,51,52]. *Streptococcus sanguinis* and *Actinomyces naeslundii* are the first bacteria to colonize the biofilm through interactions with the acquired salivary pellicle [41,53]. Immediately after the implant is exposed to the oral cavity environment, the Streptococcus sp. colonizes the implant surfaces [54,55,56,57]. These bacteria also play a major role in adhesion to organic dental substrates. The metal implant surfaces, and their local dissolution products, create a unique microenvironment that seems to dictate a distinct ecological succession [58]. For example, early peri-implant biofilms are colonized by significant lower numbers of *A. naeslundii*, and coaggregation with *Veillonella* spp. is also observed [59,60].

The microorganisms known as early colonizers will multiply, change the environment, and encourage, via co-aggregation, the adhesion of secondary colonizers [53]. A shift in bacterial biofilm composition might occur after several weeks of uninterrupted biofilm maturation. When it coincides with the breakdown of the host immune system, peri-implant mucositis may be triggered. This is also regarded as an important transitional event in the progress of peri-implant mucositis to peri-implantitis [3,61]. The transition from healthy peri-implant sulcus to a diseased peri-implant pocket is marked by the rise of Gram-negative cocci, spirochetes and motile bacilli. A shift in biofilm composition is frequently more visible in submucosal rather than supramucosal peri-implant biofilm [31,32,51,52,62,63,64].

Although peri-implant and dental biofilm share some similarities, the biofilm around an implant appears to be less qualitatively heterogeneous in microorganisms than those found on adjacent teeth [65]. In addition, peri-implant tissue morphology and structure are different from their dental counterparts. Implants show a reduced blood flow, as there is no periodontal space, and a scar is formed on the soft tissue. A deeper implant sulcus permits deeper infiltration of bacteria [66,67]. Therefore, even a completely integrated dental implant is more vulnerable to bacterial infection than periodontal tissues [5].

### 4.2. The Peri-Implant Biofilm Structure

Connectance is an important property in the structure of the bacterial community [33]. Ecosystems with higher connectance are less prone to losing hub species than systems with lower connectance [68]. They are also more stable when subjected to colonization—extinction dynamics [69]. However, when the connectance exceeds beyond a certain limit, the local balance of the community seems to diminish quickly [70].

Bacterial competitive interaction seems to be advantageous to both competitors involved. It might also enhance the fitness of the entire microbial community [71], behaving as a protective mechanism in the oral microbiome, where the establishment of exogenous species is suppressed [40]. The onset of the peri-implant inflammation interferes with the competitiveness between species in the peri-implant microbiome [33]. A significant change in the bacterial competition was found in both supramucosal and submucosal microbiome, in the transition from health to disease, not just in terms of number or proportion. Additionally, the healthy submucosa microbiome had more hub species which were sensitive to select loss. In this study, the high connectance prevented these species from detaching. However, when the tissues around the implants became inflamed, the submucosa communities became less connected and competitive, with a limited number of hub species. On the other hand, the diseased supramucosal communities were more connected and competitive than the healthy counterparts, presenting an enhanced number of hub species. These data showed that the peri-implant dysbiosis is associated with dramatic changes in the community structures, bacterial correlations, and local stability. Future studies should focus on the interaction between community structures of the submucosa microbiome and peri-implant diseases [33].

Environmental factors, such as oxygen level, pH, metabolites, nutrient availability, implant characteristics, site-specific microbiota and host response will determine which species will successfully colonize the biofilm [72,73]. Thus, the ecological conditions can accelerate shifts in the behavior and composition of the endogenous microbiota (bacterial pathogens growth) that might be unbearable for host tissues [32]. All the factors involved in the shift from a healthy to a disease-associated oral microbiome are still not completely known. To date, some conditions can be associated with the microbiological transition of oral biofilms, triggering the overgrowth of putative species with pathogenic ability. These include for example, poor oral hygiene [74], the inflammatory process [31], lack of regular dental implant maintenance [75], frequency of sucrose intake [76], products disseminated by implant deterioration [77], and nature of the EPS-enriched environment [78]. In addition, there is an individual predisposition, as determined by individual distinctive biological factors that will determine disease onset, activity, and progression [79].

## 5. Peri-Implant Microbial Profile

Even though microbiome diversity and richness differ in the literature, recent studies have reported that a shared basis of microbiota with a characteristic structure with respect to health and disease might exist. A conversion from healthy peri-implant sulcus into an inflamed peri-implant pocket is connected to subject-specific bacterial changes in peri-implant biofilm [5,14]. These may also be associated with the patient’s overall health condition, geographical and environmental characteristics, smoking and diet [5]. The peri-implant sulcus/pocket microbial composition presents a similar level of taxonomic diversity and community structure, and an elevated number of shared taxa, but in distinct quantities [22]. Early investigations of peri-implant microbiota relied on culture analyses and darkfield microscopy [22,80,81,82].

These studies provided the initial insight to better understand the microbiota associated with peri-implant health and disease. However, some oral bacteria require specific conditions or media for growth, or cannot be cultured. The advance in molecular techniques have generated a significant data body that enabled the characterization of the microbial diversity of peri-implant biofilms [22,83,84,85,86]. The current known peri-implant microbial profile for health, mucositis and peri-implantitis conditions (Table 1) can be described as follows:

(a) Healthy implant microbiome profile

The peri-implant health microbiota have been described as predominantly Gram-positive cocci and non-motile bacilli, with low counts of Gram-negative anaerobic species [47,81,87]. The core microbiota associated with health implants still need to be established in future studies. However, some studies have already identified some bacteria species that seem to be associated with healthy conditions. Few studies have focused on the peri-implant supramucosal microbiome, showing that these communities are colonized with *Prevotella multiformis*, *Kingella oralis*, *Actinomyces massiliensis*, and *Lautropia mirabilis.* These bacteria are known as the principal contributors of negative correlations in healthy communities [33]. On the other hand, studies on the peri-implant submucosa microbiome are more common in the literature, as they are used as control in research studies. The microorganisms identified and associated with the peri-implant submucosa microbiome are the following: *Streptococcus* sp. [22,88,89], *S. sanguinis* [33,90], *Streptococcus salivaris* [22], *Streptococcus oralis* [33], *Actinomyces* [22,89,91], *A. naeslundii* [44,92,93], *Actinomyces oris* [44,92,93], *Actinomyces meyeri* [44,92,93], *A. massiliensis* [33], *Veillonella* spp. [91], Veillonella dispar [90], *Rothia* sp. [44,92,93], *Rothia dentocariosa* [90], *Rothia aeria* [22,33], *Prevotella melaninogenica* [22], *Leptotrichia wadei* [22], *Mycoplasma salivarium* [22], *Neisseria* sp. [44,92,93], *Haemophilus parainfluenzae* [33], *Corynebacterium matruchotii* [33], *Leptotrichia hofstadii* [33], *Capnocytophaga sputigena* [33,94], *Eikenella corrodens* [94] and *Fusobacterium* sp. [94]. However, there is a lack of standardization in the peri-implant oral microbiome, and the studies that specified the supramucosa and submucosa microbiota associated with health peri-implant sites are summarized in Table 1. The healthy peri-implant sulcus (without any symptoms of inflammation) is also colonized, in low levels and proportions, by periodontal pathogens such as *Aggregatibacter actinomycetemcomitans*, *Porphyromonas gingivalis*, *Fusobacterium nucleatum*, *Tanerella forsythia*, *Treponema denticola*, *Prevotella intermedia*, *Parvimonas micra* and *Streptococcus intermedius* [44,91,95,96,97,98,99,100,101,102,103]. Despite the presence of periodontal pathogens, in individuals with stable periodontal condition and good oral hygiene, dental implants have successful treatment outcomes without infection [96,103].

Some studies have identified a greater number of bacteria belonging the classes Actinomycetia [90], Bacilli (genus *Granulicatella*), Gammaproteobacteria (genus *Vibrio*), and Epsilonproteobacteria (genus *Campylobacter*), in peri-implant health sites [104], while the genus *Filifactor* (commonly detected in sites with the so-called chronic periodontitis), *Bradyrhizobium*, *Dialister*, *Paludibacter*, *Staphylococcus*, *Acinetobacter*, *Propionibacterium* and *Mogibacterium* were only found at healthy sites [59].

Zhang et al. 2022 [33] described some interactions common to all communities, regardless of sampling sites or health status. This shared structure was mostly built by the phyla *Firmicutes*, *Spirochaetes* and *Bacteroidetes* species. Unlike the distinctive negative interactions which determine the microbiome health status, these shared interactions seem to be continuous, and might lead to a primary framework for peri-implant microbiome.

(b) Peri-implant disease

Peri-implant mucositis and peri-implantitis are nonspecific, heterogeneous and polymicrobial diseases of an endogenous nature [5]. Studies dealing with the microbiota associated with peri-implant disease have been extensively published. For didactic purposes, the microorganism profiles reported in the literature for peri-implant disease were divided into peri-implant mucositis and peri-implantitis:

**Table 1:** Most prevalent microorganisms identified in the supramucosal and submucosal microbiota associated with peri-implant health and disease.

	Supramucosal	Submucosal
Peri-implant Health [22,33,44,58,88,89,90,91,92,93,94,104]	*A. massiliensis* *K. oralis* *L. mirabilis* *P. multiformis*	*A. massiliensis* *A. meyeri* *A. naeslundii* *A. oris* *Actinomyces* sp. Actinomycetia class Bacilli class (genus *Granulicatella*) *C. sputigena* *C. matruchotii* *E. corrodens* Epsilonproteobacteria class (genus Campylobacter) *Fusobacterium* sp. Gammaproteobacteria class (genus Vibrio) genus *Acinetobacter* genus *Bradyrhizobium* genus *Dialister* genus *Filifactor* genus *Mogibacterium* genus *Paludibacter* genus *Propionibacterium* genus *Staphylococcus* *H. parainfluenzae* *L. hofstadii* *L. wadei* *M. salivarium* *Neisseria* sp. *P. melaninogenica* *R. aeria* *R. dentocariosa* *Rothia* sp. *S. oralis* *S. salivaris* *S. sanguinis* *Streptococcus* sp. *V. dispar* *Veillonella* sp.
Peri-implant mucositis [89,105,106,107,108]	-	*A. gerencseriae* *C. rectus* *C. ochracea* *D. pneumosintes* *Fusobacterium* sp. Genera *Fusobacterium* Genera *Prevotella* *P. micros* *P. gingivalis* *P. denticola* *P. intermedia* *T. forsythia* *T. denticola*
Peri-implantitis [18,22,23,31,58,72,81,83,90,95,104,107,108,109,110,111,112,113,114,115,116,117,118,119,120,121]	-	Actinobacteria class (genus *Micrococcus*) *A. actinomycetemcomitans* Bacteroidia class *Candida* sp. *C. leadbetteri* *Chloroflexi* spp. Clostridia class (species *Catonella morbi* and *Clostridiales* spp. HOT-093) Deltaproteobacteria class *D. invisus* *E. aerogenes* *E. cloacae* Epstein–Barr virus *E. coli* *E. saphenum* *F. alocis* *F. fastidiosum* *Fretibacterium* HMT 360 *F. nucleatum* Gammaproteobacteria (genus *Moraxella* and *Acinetobacter*) class *H. pylori* Human cytomegalovirus *L. lactis* *Mitsuokella* spp. HOT 131 *Neisseria* sp. *Peptostreptococcus* sp. *P. endodontali* *P. gingivalis* *P. nigrescens* *P. oris* *Porphyromonas* spp. HOT-395 *P. intermedia* *P. aeruginosa* *Pseudomonas* sp. Spirochaetes *S. aureus* *S. epidermidis* Synergistia class (species *Syergistetes* spp. HOT-360) *T. forsythia* *Tenericutes* spp. *T. denticola* *T. maltophilum*

b.1. Peri-Implant Mucositis Microbiome Profile

Peri-implant mucositis microbial communities are intermediate between peri-implant healthy and peri-implantitis sites [25,89,105]. When a progressive switch in the peri-implant microbiota from peri-implant health to peri-implant mucositis occurs, a higher presence of cocci, motile bacilli and spirochetes is identified [47,91,105].

Higher numbers of periodontal pathogens (*P. intermedia*, *P. gingivalis*, *T. forsythia*, *T. denticola* and *Prevotella denticola)* were found at peri-implant mucositis sites [89,105]. Emrani et al. (2009) [106] published a case report evaluating the submucosal biofilm of a patient with previous severe periodontitis, before and after full-mouth implant-supported prosthesis treatment. Microbiological culture of the biofilm of peri-implant mucositis sites showed Gram-negative facultative enteric rods and periodontal pathogen species, including *Fusobacterium* species, *P. gingivalis*, *T. forsythia*, *P. intermedia*, *Campylobacter rectus*, *Dialister pneumosintes* and *Peptostreptococcus micros*. They resembled the microorganisms collected from teeth with periodontal disease before extraction from the same patient, showing the presence of Gram-negative facultative enteric rods, *Fusobacterium* species, *P. gingivalis*, *T. forsythia*, *C. rectus*, *P. micros* and *D. pneumosintes* [106].

Recently, Zhou et al. (2022) [89] investigated the bacterial diversity in peri-implant biofilm and the effect of previous periodontitis on the occurrence of peri-implant mucositis. The authors found a bigger risk of peri-implant mucositis when an increased accumulation of *Prevotella* and *Fusobacterium* and a decrease in health-associated bacteria were identified. The authors inferred that individuals with a previous history of periodontitis may be more predisposed to develop peri-implant mucositis.

In a short-term clinical study, plaque samples from healthy sites were correlated to those with peri-implant mucositis and peri-implantitis. From the 40 bacteria species quantified, only *Capnocytophaga ochracea* was increased in the mucositis group, when compared with the healthy and peri-implantitis groups. The number of *Actinomyces gerencseriae* was higher in the mucositis group when compared with the peri-implantitis group [107]. Recently, Zhou et al. (2022) [89] reported that the genera *Prevotella* and *Fusobacterium* could act as possible biomarkers for peri-implant mucositis.

It seems that the peri-implant mucositis microbiome profile is intermediate between those of healthy and peri-implantitis sites [105]. In fact, the microbiota related to peri-implant mucositis seem to be almost identical to the ones related to peri-implantitis [94,107]. It must be considered that most studies focused on peri-implantitis, or that the microbiome of peri-implant mucositis sites were neglected or confounded with peri-implantitis [58,89,108]. To better understand the shift that occurs in the microbiome from health to disease, the peri-implant mucositis, the microbial profile needs to be clarified. More studies are needed to identify the core microbiota related to peri-implant mucositis. The studies that specified the supramucosal and submucosal microbiota associated with peri-implant mucositis sites are represented in Table 1.

b.2. Peri-Implantitis Microbiome Profile

Peri-implantitis is characterized by bone loss around the implant and the formation of a deepened peri-implant pocket, creating a habitat with a low-oxygen condition. This change in habitat is unfavorable to the growth of aerobic bacteria [47]. A complex peri-implant biofilm community is then formed, and it is composed of different species of bacteria, fungus, and virus, distributed according to their biochemical and nutritional needs [7,18,31]. The first anaerobic cultures and phase-contrast microscopy studies detected Gram-negative, motile, black-pigmented and anaerobic bacteria species at the onset of periimplantitis, also known to be widespread in periodontitis [31,81,91]. The studies that specified the supramucosa and submucosa microbiota associated with peri-implantitis sites are represented in Table 1.

Formerly, there was an assumption that the composition of the peri-implantitis submucosal microbiome was similar to that found in periodontitis, with a mixed anaerobic infection dominated by Gram-negative bacteria, including the periodontal pathogens *A. actinomycetemcomitans*, *Capnocytophaga leadbetteri*, *F. nucleatum*, *P. intermedia*, *T. forsythia*, *T. denticola*, *Treponema maltophilum* and *P. gingivalis*, as they were identified in peri-implantitis samples [22,23,58,72,81,83,90,95,107,108,109,110,111,112,113].

More recently, a higher number of other microbes not usually connected to periodontal diseases, such as *Staphylococcus aureus*, *Staphylococcus epidermidis*, *Pseudomonas aeruginosa*, *C. leadbetteri*, Peptostreptococcus sp., Neisseria sp., *Porphyromonas endodontali*, *Lactococcus lactis*, *Filifactor alocis*, *Escherichia coli*, *Enterobacter aerogenes*, *Helicobacter pylori*, *Enterobacter cloacae*, Pseudomonas species and Candida species, were also identified in peri-implantitis sites [18,22,23,31,97,114,115,116,117,118,119,120]. Some viruses have also been associated with peri-implant infection, such as Epstein–Barr virus (EBV) and Human cytomegalovirus (HCMV). These viruses might play an etiologic role by suppressing the local immune system, allowing the overgrowth of periodontopathogens, with a reported co-infection rate of 33% of the peri-implantitis sites [121].

As technology advances, more species can be identified and quantified in peri-implantitis sites. For example, a higher number of bacteria belonging to the Gammaproteobacteria (genus *Moraxella* and *Acinetobacter*), Actinobacteria (genus *Micrococcus*) [104], Spirochaetes, Synergistia (species *Syergistetes* spp. HOT-360), Bacteroidia, Deltaproteobacteria, Clostridia classes (species *Catonella morbi* and *Clostridiales* spp. HOT-093) has been detected. Other bacterial species such as *Porphyromonas oris*, *Porphyromonas* spp. HOT-395, *Porphyromonas nigrescens*, *Dialister invisus*, *T. maltophilum*, *Freitbacterium fastidiosum*, *F. alocis*, *Eubacterium saphenum*, *Chloroflexi* spp., *Mitsuokella* spp. HOT 131, *Fretibacterium* HMT 360 and *Tenericutes* spp. [90] have also been reported. Nevertheless, there is still an absence of consensus in relation to the etiopathogenesis of peri-implantitis, as other studies could not identify the association of some of these bacteria with peri-implantitis [23].

Other studies had conflicting results regarding bacterial diversity and community structure, when comparing peri-implantitis to control (peri-implant healthy) implant sites [22,57,59,90,104,105,111,113,122,123,124]. Although some studies found remarkable similarities in peri-implantitis sites and control implants [22,57,123,124,125], other studies suggested that peri-implantitis microbiota are more heterogenous than control implants [90,104,105,113]. It is important to note that in the literature, the diagnostic criteria of peri-implantitis is quite heterogenous; therefore, it can influence the results of the microbiome analysis. This fact might explain the different predominant peri-implantitis core microbiome species identified in the literature [22]. The divergent results could also derive from subject, diagnostic, experimental, and bioinformatic differences among studies [21,122]. At the experimental level, the DNA extraction protocol can also impact considerably the microbiome results [126]. Future studies are needed to clarify the peri-implantitis core microbiome.

## 6. The Interaction between the Host Immune System and the Peri-Implant Microbiota

In order to elucidate the interplay between the host immune system and the peri-implant microbiota, basic concepts involving the host-response will be briefly reviewed. The innate immunity is the first line of defense and includes saliva, antimicrobial peptides, and immune cells (macrophages and neutrophils). It is the immediate response, and is not specific to a particular pathogen. As such, the macrophages and neutrophils can recognize common microbial patterns and respond quickly to eliminate them [127,128,129,130,131]. On the other hand, the adaptive immunity is characterized by the ability to remember and specifically attack pathogens it has previously encountered. The lymphocytes are responsible for the production of antibodies (B cells) and cellular immunity (T cells). This type of immune response can take several days to initiate a response after the initial exposure to a pathogen; however, the subsequent responses are faster and more effective because of the memory cells [127,128,129].

Although peri-implant disease is known as a bacterially driven infection, the interaction between the immune system and peri-implant biofilms is a determinant factor in sustaining dental implant health. It also plays a role in peri-implant disease pathogenesis [5,14]. The resulting dysbiosis and sustained inflammation, which promotes peri-implantitis progression, enters a vicious cycle, whereby one potentializes the severity of the other [130]. This relationship seems to be a dynamic process entailing both innate and adaptative immune responses [129,130,131].

The migration of polymorphonuclear leukocytes through connective tissue is mediated by bacterial products. During this initial phase of the inflammatory process, microvascular changes (vasodilation) and pro-inflammatory cytokine release are observed [5]. However, the peri-implant biofilm can trick the host immune system by creating physical barriers that prevent immune cells from entering and antimicrobial substances from being efficacious. Persistent oral biofilms can lead to chronic inflammation [127].

The steady effort of the host immune system to eliminate the peri-implant biofilm increases the vasodilation and vasoproliferation, and activates the inflammatory response moderated by the activation of innate immune cells (macrophages, dendritic cells, and mast cells) [5,127]. Neutrophils stimulate the liberation of pro-inflammatory cytokines, such as interleukin-1 (IL-1) and Tumor Necrosis Factor alpha (TNF-α), which in turn activate osteolytic and inflammatory tissue damage [132]. A higher concentration of IL-1b, interleukin-8 (IL-8), TNF-α, tissue inhibitor of metalloproteinase-2 (TIMP-2), vascular endothelial growth factor (VEGF), and osteoprotegerin (OPG) have been reported in the peri-implantitis crevicular fluid, in comparison to healthy implants [14,133,134]. Tissue samples collected from sites with periimplantitis also expressed higher levels of interleukin-6 (IL-6), IL-8, and TNF-α when compared to healthy sites [135]. Defects in neutrophil efficacy or number can put individuals at risk of periodontal disease. Paradoxically, neutrophil activity, as part of a deregulated inflammatory response, seems to be a key factor in the destructive disease process [136].

The C-X-C Motif Chemokine Receptor 2 (CXCR-2), also known as Interleukin 8 receptor beta (IL8RB), is an essential stimulant of immune cell migration and recruitment, mainly expressed on neutrophils [137]. The lack of CXCR-2 neutrophil receptor in the gingival tissue has been connected to changes in the local microbiome, causing the exacerbation of the periodontal disease. This fact illustrates the neutrophils’ important role in the maintenance of a balanced oral microbiota. In addition, the gingival tissue microbiota were reestablished when active CXCR-2 neutrophil receptors were present [136]. Although this study was performed on teeth with periodontal disease, it leads to key information which can help clarify the mechanism of peri-implant diseases. Further studies evaluating the CXCR-2 role in peri-implant disease are still needed.

Concurrently, macrophages might exert a dual function in directing implant success or failure, depending on their phenotype [132]. Two distinct functional phenotypes can be produced by macrophages: M1 or M2 (macrophage polarization). The balance between M1 and M2 macrophages strongly affects the progression of inflammatory disorders [138]. The M2 macrophages have been associated with successful wound healing and osseointegration, while M1 macrophages seem to exacerbate the inflammatory process and accelerate osteolysis, resulting in dental implant failure. Significantly, a higher M1 profile was associated with advanced peri-implantitis when compared with M2 expression [139,140].

Inflammasomes are multiprotein complexes induced by diverse inflammatory stimuli [141]. They mediate the caspase-1 activation, promoting the production of the proinflammatory cytokines interleukin 1β (IL-1β) and IL-18 [141]. Components of the Nod-like receptor family, such as NLRP1, NLRP3 and NLRC4, and the adaptor ASC are key parts of the inflammasome. They link microbial and endogenous ‘danger’ signals to caspase-1 activation. Several diseases are associated with dysregulated activation of caspase-1 and secretion of IL-1β [141] and the peri-implant inflammation seems to be one of them [142]. A pioneer cross-sectional study recently demonstrated that inflammasomes AIM2 and NLRP3, and their downstream effectors interleukin-1β and caspase-1, are strongly associated with specific bacteria in peri-implantitis [142].

Inflammasomes can also mediate pyroptosis, a form of cell death caused by bacterial pathogens [141]. Pyroptosis is a driving factor of local inflammation, bone resorption, and in situ collagen breakdown [143]. It is associated with plasma membrane leakage, which leads to inflammation of the surrounding tissue through liberation of pro-inflammatory mediators [144]. Pyroptosis can occur as a response to the presence of bacterial antigens, the slow continued release of bacterial metabolism by products, exotoxins, endotoxins (in particular, lipopolysaccharides released by Gram-negative bacteria) [143], and even as a response to mechanical stress per se, factors which can all lead to the assembly of inflammasomes, thereby initiating pyroptosis [144]. Caspases are the most important effectors of pyroptosis, and their role in peri-implantitis is the focus of several ongoing studies [143].

During bacterial infection, inflammasomes are activated, leading to uncontrolled bone resorption. This unrestricted inflammasome activity leads to osteolysis of the alveolar bone, through the activation of neutrophils, monocytes, macrophages, and adaptive immune cells, such as T helper 17 cells. The immune response generates a growth in osteoclasts and a concomitant decline in osteoblasts. In addition, osteocytes play a decisive role in alveolar bone loss, which occurs as a response to these inflammatory changes, by secreting molecules that affect bone resorption and formation [145].

## 7. Role of Implant Surface on the Microbiota of Peri-Implantitis

Dental implants and teeth differ in several key structural aspects, including morphology, surface roughness, energy and material [146]. Although little information is available on the effect of different types of implant surfaces and implant materials on the peri-implant microbiome, this topic has recently been studied [147].

Surface roughness impacts both osseointegration and biofilm formation [148]. Most implants currently in use are engineered with moderately rough surfaces, with roughness levels ranging from 1 to 2 μm [149], with the bacterial retention threshold being 0.2 μm. Above this level, an increase in bacterial accumulation occurs [148]. Establishing the ideal surface roughness for implants is extremely difficult. To secure a powerful connection between bone and implant, a minimum surface roughness of 1 to 1.5 micrometer is required. However, surface roughness higher than 0.2 micrometer can enhance bacterial adhesion. Balancing antimicrobial capabilities and beneficial osteoconductive environment is extremely difficult. While greater roughness level improves bone integration, it also favors bacterial adherence, possibly stimulating biofilm growth [148].

Surface roughness and surface free energy have been identified as important determinants of biofilm composition in the peri-implant sulcus [150,151]. In vitro investigations showed that *Streptococcus pyogenes*, *P. gingivalis*, *A. naeslundii*, *Streptococcus mutans*, *Lactobacillus salivarius* and *F. nucleatum* adhered better to roughened titanium, either acid-etched or sand-blasted surfaces [152,153,154,155,156,157]. Other studies did not find significant differences in microbial composition around diseased implants with different surfaces [158,159]. However, these studies were performed on dogs, and limited to targeted pathogens that are not representative of natural pathobionts in that animal model [160].

Additional modifications of the implant surface are often incorporated to enhance osseointegration [149,161,162,163] and to interfere with microbial adhesion, playing an important role in the biofilm formation [161]. They include machining, sand blasting, acid-etching, sintering, oxidizing, plasma-spraying, hydroxyapatite coating, laser-modification, or a combination of these procedures, to modify the implant surface [163].

A recent cross-sectional clinical study [160] evaluated the influence of different implant surface topographies (anodized surface [AN], sandblasted acid-etched surface [SLA], and hydroxyapatite-coated surface [HA]) on the peri-implant microbiome. The peri-implant microbiome was characterized and quantified in both healthy and diseased conditions. The study found that the microbiome in peri-implant health is not influenced by different implant characteristics when the implant is placed at or below the level of the alveolar crest and is sealed from the peri-implant sulcular environment by a soft-tissue attachment apparatus. In this case, modifications to the surface of the implant body would not impact microbial colonization in the sulcus. The location of the implant–abutment connection, the material, and the surface characteristics of the coronal structure are likely variables, and further studies are warranted [160]. In another study, anodize- and hydroxyapatite-coated implants were found to be microbially similar in health. However, in a peri-implantitis state, dysbiosis was more pronounced in hydroxyapatite-coated implants than anodize, with the loss of several health-compatible species and the enrichment of over 40 others. Although there are limited commonalities among the enriched species in peri-implantitis across different implant types, it is evident that most enriched species in peri-implantitis-associated implants belong to well-known oral pathogenic genera. An interesting finding was the lack of difference between healthy and peri-implantitis implants in sandblasted acid-etched modified implants. While it is possible to attribute this to the small sample size in this group (10 healthy implants and 16 implants with peri-implantitis), previous studies of a similar sample size found differences between peri-implant health and disease [111]. The authors suggested that factors other than dysbiosis might play a role in disease initiation, concluding that surface topography is a modifier of the disease-associated peri-implant microbiome, and that the extent of this impact varies widely among the different modifications. This study underscores the multi-factorial nature of peri-implant diseases, and speculates that implant surface topography might directly or indirectly influence susceptibility to disease [160].

Nano-level chemical alterations on implant surfaces are acquired to enhance the surface’s hydrophilicity, stimulating osseointegration while diminishing hydrophobic bacterial attachment [148,164]. It has also been found that there is a reduction in biofilm growth, especially of pathogens such as *T. forsythia*, *P. gingivalis* and *T. denticola*, after 30 days of exposure when compared to other surface compositions [164]. D’Ercole et al. (2020) [165] demonstrated that nano-roughness and hydrophilicity of polyetheretherketone can significantly alter the number of bacterial Colony Formation Units (CFUs) and the biofilm mass of *S. oralis*. This illustrates a bactericidal and/or non-adhesive effect. Additionally, a carbon fiber-reinforced polyetheretherketone has been assessed as a replacement for implant titanium material. To date, no microbiological studies have been developed to verify the biofilm formation of this type of material [165]. Bright et al. (2021) [166] demonstrated a reduction in pathogenic species *P. aeruginosa* and *S. aureus*, and in their viability in a 2 μm layer furthest from the titanium nanostructured surface Ti6Al4V [166]. These can be fabricated in a variety of materials, such as titanium, zirconia, hybrid (titanium body and zirconium oxide abutment) and stainless steel [127]. The incorporation of zirconium and niobium in the titanium implant blend had similar bacterial adherence behavior compared to implants composed of solely titanium and vanadium, demonstrating a slight uptick in adhesion of *S. sanguinis* and *A. naeslundii* [167].

Conclusive studies on correlations between the implant surface and peri-implant microbiota are still lacking. More knowledge is still needed regarding peri-implantitis risk factors and the optimal implant material and surface composition which will be resistant to peri-implant diseases.

## 8. Evidence of the Titanium Particle Effect on the Peri-Implant Microbiome

Titanium is widely used in dental implants. However, some studies have reported titanium corrosion and attrition, due to implant exposure to the oral cavity conditions, and/or frictional forces at the implant–abutment interface. As a result of this, ions and metal nano- or microparticles would be liberated in the peri-implant soft tissue [168].

The initiation of peri-implantitis related to the existence of metallic particles, or their interaction with or synergistic effect on periodontal pathogens, is a matter currently under discussion [169]. It is also ambiguous as to whether this release of metallic material can induce a tissue inflammatory response and, in connection with the presence of the local microbiota, perform a crucial role in the development of peri-implant disease [170]. Additional studies are required to fully comprehend the contribution of ion/particle release in the pathogenesis of peri-implant diseases.

## 9. Similarities and Differences between Peri-Implantitis and Periodontitis

Peri-implantitis and periodontitis are clinically very similar, as both diseases are infections mediated by a dysbiotic biofilm associated with an hyperinflammatory reaction, leading to progressive alveolar bone resorption [33,171,172,173,174]. Yet, their pathogenic mechanisms appear to be different. The biofilm accumulation, followed by its microbial dysbiosis, is regarded as the initiation factor of both conditions [33,175]. On the other hand, the progression of the inflammatory destructive disease around dental implants differs from that around teeth [176].

Recently, it has been found that peri-implant and periodontal microbiomes differ greatly. The first study using next-generation sequencing techniques to compare peri-implant and periodontal microbiota showed that 85% of the subjects studied shared less than 8% of the bacteria between peri-implant and periodontal sites [111].

As previously discussed, the peri-implant microbiome hosts a unique bacterial ecology in comparison to the periodontal microbiome. It is numerally lower with regard to microbial diversity, but numerally higher for a few bacterial genera [5,105,111,177,178,179,180], regardless of health or disease status, and its complexity increases when moving from peri-implant mucositis to peri-implantitis [105,178].

Data from a single study assessing bacterial messenger RNA suggest that the inherent characteristics of the microbiota in sites with peri-implantitis and periodontitis are similar [181]. Nevertheless, the ability with respect to inter-bacterial interaction seems to be more complex at sites with peri-implantitis [181]. To gain a deeper understanding of the complex interactions between the host’s response and the peri-implant microbial biofilm, additional studies and newer models are needed [5,178,179,180].

## 10. Conclusions

Advances in molecular analysis have demonstrated that the peri-implant microbiome differs from the microbiome that surrounds teeth, in both healthy and diseased states. They differ structurally and chemically. The adhesion and formation of the peri-implant biofilm can be affected by the surface energy, topography, wettability, and electrochemical charges of the implant surface. In addition, the morphogenesis of the tissues surrounding the dental implant also diverges from that of the teeth, rendering dental implants more vulnerable to bacterial infections. The interrelation of the host immunity and the microbiome in peri-implant infections remains to be elucidated.

## 11. Future Directions

The intricate interplay of the microbiota, host and environmental factors requires greater understanding to achieve improved treatment outcomes with dental implants. Highly specialized approaches for both preventive and curative therapies in the field of dental implantology are still lacking. To be able to compare studies, the use of standardized protocols to investigate the microbiota in relation to implant health and disease is also necessary. Future research should also focus on developing comprehensive diagnostic protocols that combine radiological, clinical, molecular and microbiological patterns.

## Data Availability

No new data were created. Data sharing is not applicable to this article.
